# Orally administered *Taenia solium* Calreticulin prevents experimental intestinal inflammation and is associated with a type 2 immune response

**DOI:** 10.1371/journal.pone.0186510

**Published:** 2017-10-16

**Authors:** Fela Mendlovic, Mayra Cruz-Rivera, Jose Alfredo Diaz-Gandarilla, Marco Antonio Flores-Torres, Guillermina Avila, Maria Perfiliev, Ana Maria Salazar, Lourdes Arriaga-Pizano, Patricia Ostrosky-Wegman, Ana Flisser

**Affiliations:** 1 Departamento de Microbiologia y Parasitologia, Facultad de Medicina, Universidad Nacional Autonoma de Mexico, Ciudad de Mexico, Mexico; 2 Facultad de Ciencias de la Salud, Universidad Anahuac Mexico Norte, Huixquilucan, Estado de Mexico, Mexico; 3 Division Academica Multidisciplinaria de Comalcalco, Universidad Juarez Autonoma de Tabasco, Comalcalco, Tabasco, Mexico; 4 Departamento de Medicina Genomica y Toxicologıa Ambiental, Instituto de Investigaciones Biomedicas, Universidad Nacional Autonoma de Mexico, Ciudad de Mexico, Mexico; 5 Unidad de Investigación Medica en Inmunoquimica, Hospital de Especialidades CMN "Siglo XXI", IMSS, Ciudad de Mexico, Mexico; Case Western Reserve University, UNITED STATES

## Abstract

Intestinal helminth antigens are inducers of type 2 responses and can elicit regulatory immune responses, resulting in dampened inflammation. Several platyhelminth proteins with anti-inflammatory activity have been reported. We have identified, cloned and expressed the *Taenia solium* calreticulin (rTsCRT) and shown that it predominantly induces a type 2 response characterized by IgG1, IL-4 and IL-5 production in mice. Here, we report the rTsCRT anti-inflammatory activity in a well-known experimental colitis murine model. Mice were orally immunized with purified rTsCRT and colitis was induced with trinitrobenzene sulfonic acid (TNBS). Clinical signs of disease, macroscopic and microscopic tissue inflammation, cytokine production and micronuclei formation, as a marker of genotoxicity, were measured in order to assess the effect of rTsCRT immunization on experimentally induced colitis. rTsCRT administration prior to TNBS instillation significantly reduced the inflammatory parameters, including the acute phase cytokines TNF-α, IL-1β and IL-6. Dampened inflammation was associated with increased local expression of IL-13 and systemic IL-10 and TGF-β production. Genotoxic damage produced by the inflammatory response was also precluded. Our results show that oral treatment with rTsCRT prevents excessive TNBS-induced inflammation in mice and suggest that rTsCRT has immunomodulatory properties associated with the expression of type 2 and regulatory cytokines commonly observed in other helminths.

## Introduction

Epidemiological and experimental studies have shown that there is an inverse association between inflammatory diseases and helminth infection, thus suggesting that helminth exposure may be implicated in a lower incidence of inflammatory disorders [1, 2]. Helminths are considered “master regulators” since they are able to induce a modified type 2 immune response characterized by the activation of several components that include molecules such as IL-4, IL-5, IL-13 and the regulatory cytokines IL-10 and TGF-β. This type of response allows the persistence of the parasite in its host without overt symptomatology and is a result of coevolution [3]. The immunomodulatory properties of helminths seem to be conserved among several phylogenetic groups such as nematodes, trematodes and cestodes. Experimental infections with schistosomes and several nematode parasites result in diminished immunopathology in different models of inflammatory diseases, including colitis [4, 5]. The effect of cestode infections or their products has been less studied. Only two cestode infections, *Hymenolepis diminuta* and *Taenia crassiceps* have been shown to modulate the severity of colitis [6, 7]. Crude extracts or soluble excretion/secretion products (E/S) from other cestodes as well, have been shown to inhibit inflammation or interact with cells and induce tolerogenic phenotypes. For example, E/S from *Echinococcus spp*. and *Taenia spp*. inhibit murine dendritic cell function and induce subsequent development of T regulatory cells [8–10].

The etiology of inflammatory bowel diseases (IBD) is currently unknown. It is suggested that the pathology is the result of an exacerbated inflammatory response associated with the dysregulation of the cytokine network [11, 12]. IBD are characterized by the expression of pro-inflammatory cytokines, which are partly responsible for the initiation and propagation of the inflammatory process and act as colitogenic mediators. Moreover, inflammatory cytokines have been shown to play a significant role in mediating systemic DNA damage that result in micronuclei formation as biomarkers of genotoxicity. In particular, intestinal inflammation has been shown to elicit systemic genotoxicity that correlates with clinical symptoms as well as pro-inflammatory cytokine levels [13, 14]. In addition, cytokines affect the mucosal barrier function and recent evidence shows that defects at this level also contribute to the pathogenesis of colitis [15].

Purification of individual single proteins has proven difficult and many of the components of E/S products that possess immunomodulatory properties are glycoconjugates and are difficult to produce in the laboratory [16, 17]. Therefore, it would be useful to identify recombinant helminth proteins with immunomodulatory properties and analyze their capacity to mimic the regulatory effects of experimental infection and help dissect their different modes of action.

Calreticulin is a multifunctional protein involved in diverse cellular and immunological processes and is a component of the E/S products of several helminths [18–20]. We have previously described the ability of orally administered recombinant *Taenia solium* calreticulin (rTsCRT) as a candidate vaccine to reduce worm burden in a hamster model of taeniosis by inducing a IL-4 and hyperplasia of goblet cells in the small intestine and to reduce the DNA damage brought about by the presence of the tapeworms [21, 22]. rTsCRT was also able to induce production of IL-4 and IL-10 when incubated *in vitro* with mesenteric lymph node and spleen cells [23]. Therefore, we hypothesized that oral immunization with rTsCRT might promote an immunomodulatory environment that prevents the development of colitis using the model of 2, 4, 6-trinitrobenzene sulfonic acid (TNBS)-induced acute colonic inflammation. We followed the same dose and administration scheme that was shown to induce a predominant type 2 response with the induction of IgG1 and IgA in the serum and intestinal flush respectively and the production of IL-4 and IL-5 in mesenteric lymphoid cells of rTsCRT-orally immunized mice [24]. Several animal models of intestinal inflammation have contributed to our understanding of the underlying immunological factors involved in the inflammatory processes during acute and chronic phases of IBD. TNBS colitis has been widely used in the study of immunological mechanisms related to acute inflammation, since several characteristics such as cytokine secretion profiles, histopathology and effects of potential immunotherapies resemble the disease in humans [25, 26].

Here we report that oral immunization with rTsCRT without the addition of adjuvant before the induction of colitis with TNBS reduces the severity of colonic inflammation, the disease index and genotoxicity caused by TNBS treatment, suggesting that a single helminth-derived protein is able to ameliorate disease signs in an acute model of colitis. We propose that rTsCRT mimics the beneficial effects on the immune system that result from exposure to intestinal helminths and suggest that rTsCRT shares the immunomodulatory properties observed in other helminths.

## Materials and methods

### Ethical considerations

The Institutional Research and Ethics Committee of the Faculty of Medicine, National University of Mexico (UNAM) approved all animal procedures in accordance with the Mexican Official Guidelines (NOM-062-ZOO-1999) that are in strict agreement with recommendations in the Guide for the Care and Use of Laboratory Animals of the National Institutes of Health (USA). Experimental protocol: 081–2012. Mice were bred at the Instituto Nacional de Pediatria and cared by the team under the supervision of Dr. Ramon Garcia, Head of the Animal Facility. All efforts were made to minimize animal suffering. We used the minimal number of experimental animals per group; 5 mice were housed in stable groups in pathogen-free conditions in a temperature-controlled room (24°C), on a 12h/12h light cycle with food and water *ad libitum*. Cages were routinely cleaned, and mice were handled with care, cupped in the hand whenever possible. The mortality rate in the group treated with TNBS was 15.4% and in the group with rTsCRT + TNBS 7.7%. A weight loss of >20% was considered as a criterion for humane endpoint for animals that became severely ill prior to the termination of the experiment; no mice reached this criterion.

### Expression and purification of rTSCRT

The cDNA of TsCRT was cloned and expressed as previously described [27]. BL21 bacteria expressing rTsCRT were sonicated in 20 mM Tris-HCl buffer, pH 7.3 with protease inhibitors (Complete, Roche, Indianapolis, IN, USA) and centrifuged. The supernatant containing rTsCRT was submitted to 10% SDS-PAGE and the 53 kDa band was electroeluted. Endotoxins were measured using the Endpoint Chromogenic LAL assay kit resulting in levels < 0.1 endotoxin units/ml (Lonza, MD, USA). Protein concentration was determined by the Lowry method [28]. Purified rTsCRT was kept at -20°C until use.

### rTsCRT oral immunization and induction of colitis

Female Balb/c mice (8 weeks) were divided in 5 groups and orally immunized 4 times at weekly intervals; two independent experiments were performed, n = 11–13 mice. In the TNBS-treated group two mice died, while only one mice died in the rTsCRT + TNBS group. Group 1 and 3 were sham-immunized with 200 μl of 0.2 M bicarbonate buffer supplemented with 0.3% glucose w/v (BGB, pH 9.6) and groups 2 and 4 received 50 μg rTsCRT in 200 μl BGB without any adjuvant through oral gavage using a flexible catheter. Experimental colitis was induced in groups 3 and 4 by intrarectal administration of 150 μl of a 4 mg TNBS solution in 50% ethanol (Sigma Chemical Co., St. Louis, MO, USA) 4 days after the last immunization using a flexible catheter, as previously described [29]. Groups 1 and 2 received 150 μl of phosphate buffer saline (PBS, pH 7.4) instead of the TNBS. An additional group received 150 μl of a 50% ethanol solution as a control of toxic reaction [30]. For intrarectal manipulation, mice were fasted for 16 h with free access to water and anesthetized by inhalation of sevofluorane (Svofast, Baxter Int., Deerfield, IL). Afterwards, animals were replaced in their cages with free access to food and water. On day 3 after TNBS instillation, mice were euthanized by an overdose of sevofluorane (Svofast, Baxter Int., Deerfield, IL).

### Clinical evaluation of mice

The clinical evaluation of the inflammatory reaction in mice was performed daily according to the following parameters: a) weight loss (no loss = 0 point (pt); <10% = 1 pt; >10% = 2 pt); b) consistency of stools/diarrhea (normal = 0 pt; pasty stools that do not stick to anus, no bleeding = 1 pt; diarrhea that sticks to anus, bleeding = 2 pt); c) piloerection (absent = 0 pt; moderate = 1 pt; severe = 2 pt); d) inactivity (absent = 0 pt; moderate = 1 pt; severe = 2 pt) The sum of the parameters resulted in the disease index score, ranging from 0 (unaffected) to 8 points (severe disease).

### Histology

At 3 cm from the anus, one colon segment (0.5 cm) was collected for histopathological analysis from all animals. The tissues were fixed in 10% neutral formalin, dehydrated, embedded in paraffin, 2 μm sections were otained, stained with haematoxylin and eosin (Instant-Prov kit, Newprov, Pin-hais, Brazil) or Periodic Acid Schiff (PAS) and evaluated by light microscopy for the study of tissue inflammation according to a histological score. The following parameters were used: a) inflammatory infiltrate (normal = 0 pt; little infiltrate = 1 pt; infiltrate and high vessel density = 2 pt; extensive infiltrate, high vessel density and loss of goblet cells = 3 pt); b) infiltrated layers (no damage = 0 pt; infiltrate around crypts = 1 pt; infiltrate in mucosa = 2 pt; infiltrate in submucosa = 3 pt); c) loss of architecture (no damage = 0 pt; minimal loss of epithelial and goblet cells = 1 pt; minimal loss of crypts and extensive loss of epithelial and goblet cells = 2 pt; extensive loss of crypts, epithelial and goblet cells = 3 pt); d) edema (absent = 0 pt; present = 1 pt). Scoring was performed by two investigators blinded for the experimental groups.

### Quantitative RT-PCR (qRT-PCR)

For the analysis of gene expression by qRT-PCR in colonic and mesenteric lymph nodes (MLN), total RNA was isolated using Trizol (Invitrogen, Carlsbad, CA, USA) following the manufacturer’s protocol. All RNA samples were quantified with the Thermo Scientific NanoDrop 2000 Spectrophotometer (Thermo Scientific, Wilmington, DE, USA) and 3 μg of RNA were submitted to reverse transcription using oligo (dT) primers with the Superscript III First-Strand Synthesis System (Invitrogen, Carlsbad, CA, USA). A Taqman gene expression assay was performed using commercially available primers and probes (Applied Biosystems, Austin, TX, USA) for IL-4 (Mm00445259_m1), IL-10 (Mm00439614_m1), IL-13 (Mm00434204_m1), IL-1-β (Mm01336189_m1), IL-6 (Mm00439653_m1), TNF-α (Mm00443258_m1) and TGF-β (Mm01178820_m1) on a LightCycler 2.0 (Roche, Indianapolis, IN, USA) in a 10 μl reaction volume containing 3 μl cDNA, 2 μl TaqMan Universal PCR master mix (Roche, Indianapolis, IN, USA), 0.5 μl Taqman assay probe and 4.5 μl RNase-free H_2_O. The parameters for PCR amplification were 95°C for 10 min, followed by 55 cycles each consisting of denaturation at 95°C for 10 s, annealing at 60°C for 10 s and extension at 72°C for 10 s. The housekeeping gene β-actin (Mm00607939_s1) was used to normalize mRNA expression. Relative RNA quantitation was calculated using the ΔΔCt method [31].

### Cytokine determination by ELISA

Mice were anesthetized by inhalation of sevofluorane and blood was obtained by cardiac puncture. ELISA kits for IL-10 and TGF-β were used according with the manufacturer’s instructions (eBioscience, San Diego, CA) based on standard curves, serum concentrations were measured.

### Determination of micronuclei frequency by flow cytometry

Flow cytometry for micronuclei (MN) in reticulocytes (RET) was performed according to Balmus with some modifications [32]. Peripheral blood (25 μl) from mice of each group was placed into vials containing heparin and subsequently fixed with 500 μl of methanol at -20°C by vortexing for homogenous fixation for 1 min and stored at -70°C for at least 24 h. For MN staining, samples were thawed and washed with 1000 μl bicarbonate buffer (BCB, pH 7.5) twice to remove heparin and methanol. Cell counts were adjusted to 4 x 10^6^ for each sample. Cells were centrifuged at 3000 rpm for 8 min at 4°C and 25 μl of RNAse, at a concentration of 1.2 mg/ml (Thermo scientific), were added. Cells were incubated at 37°C for 50 min to remove RNA [33]. Subsequently, cells were washed with 100 μl of BCB and centrifuged under the same conditions as described above. Anti-CD71-FITC antibody (BioLegend) was added at a concentration of 1 μg/100 μl and incubated for 30 min at 4°C with slight shaking to ensure a homogenous reaction of the antibody with its receptor. Propidium iodide (50 μg/ml, Sigma Aldrich) was then added and incubated at 4°C for 25 minutes. Cells were washed with 150μl of BCB, centrifuged and 500 μl of BCB were added for analysis. Flow cytometry analysis was performed using a FACS Canto II with a FACS Diva Version 6.1 software (BD Biosciencies, San Jose California, USA). Approximately 520,000 events were scored per mouse, so as to reach approximately 10,000 positive events corresponding to CD71+ RET. The micronucleated reticulocyte (MN-RET) frequency was calculated using absolute events from the different quadrants obtained from the FlowJo LLC software using the following formula: % MN-RET = [MN-RET/(RET + MN-RET)] x 100 as described [32].

### Statistical analysis

The graphs and statistical analysis were performed using the Prism 6.0 software (GraphPad Prism, La Jolla, CA). The Kolmogorov-Smirov test was used to verify the normality of all the data. Statistical significance was calculated using Kruskal-Wallis one-way analysis of variance followed by Dunn’s post hoc test or ordinary one-way or two-way ANOVA followed by the Tukey post hoc analysis as appropriate. Mann-Whitney test was performed for comparison between two groups. Correlations tests were performed according to Spearman’s rank correlation coefficient. All data are shown as mean ± standard error of the mean (SEM). P values <0.05 were considered significant. *P<0.05; **P<0.01; ***P<0.001; ****P<0.0001.

## Results

### Oral immunization of rTsCRT prevents colitis symptoms

**Fig 1:**
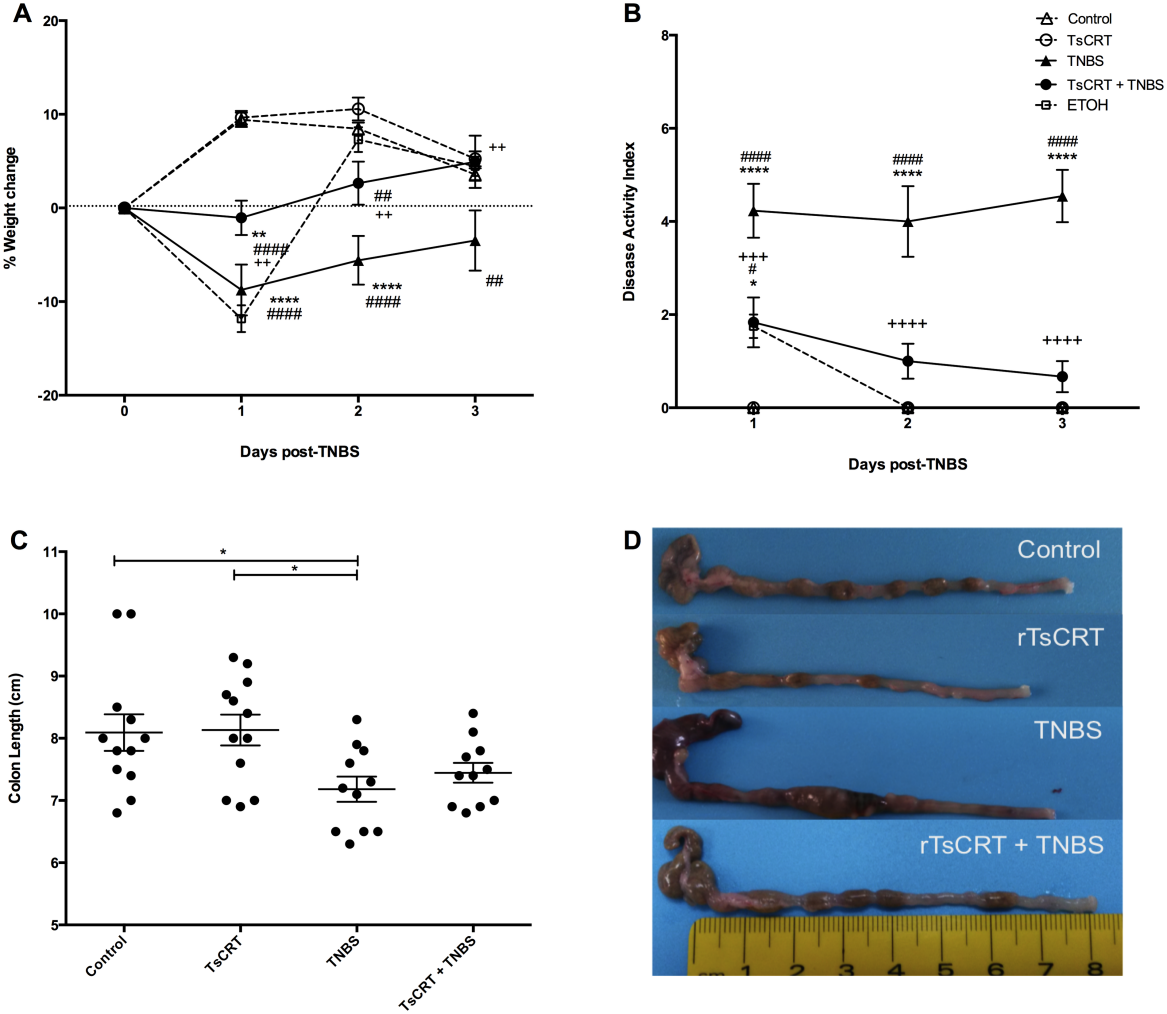
Effect of rTsCRT oral immunization on clinical parameters. Weight loss (A), disease activity index (B), colon length (C) and macroscopic characteristics of the colon (D) were evaluated 3 days after sham or TNBS treatment. Two way ANOVA was used for weight loss and disease activity index analysis and one-way ANOVA for colon length. Results are expressed as means ± SEM of pooled data from two separate experiments; n = 12,13 mice per group. *P<0.05; **P<0.01; ***P = 0.001; ****P<0.0001. Symbols: (*) as compared to control mice, (^#^) as compared to rTsCRT-immunized mice, (+) as compared to TNBS-treated mice.

TNBS inoculation resulted in the presence of clear signs of inflammation characterized by weight loss, piloerection, inactivity and bloody stools, while administration of rTsCRT without adjuvant significantly prevented weight loss and disease symptoms (Fig 1). Control and rTsCRT-immunized mice showed no weight loss or clinical disease symptoms. Mice treated with TNBS showed severe weight loss starting on day 1 after TNBS treatment and throughout the 3 days studied. Disease activity index (DAI) remained high after TNBS treatment. On the other hand, TNBS-treated mice that received rTsCRT lost only 1% weight at day 1 post-TNBS inoculation and afterwards recovered their original body weight, having at the end of the experiment the same weight as control animals. A 50% reduction in DAI with less piloerection, fecal bleeding and more physical activity was evident in this group. As expected, intra-rectal administration of ethanol, used as toxic reaction control, resulted in temporal weight loss that lasted only 24h and did not result in clinical disease (Fig 1A and 1B). Fig 1C and 1D show data and images of the large intestines in the different groups. Colon shortening and internal bleeding were signs of TNBS-induced colitis. Colons were shorter in mice that received TNBS compared with those of control and orally treated mice. Previous immunization with rTsCRT had no significant effect on the intestine shortening induced by TNBS (Fig 1C). However, rTsCRT administration prevented intestinal bleeding and macroscopic inflammation (Fig 1D). No macroscopic damage was observed in control, rTsCRT- or ethanol-treated mice.

**Fig 2:**
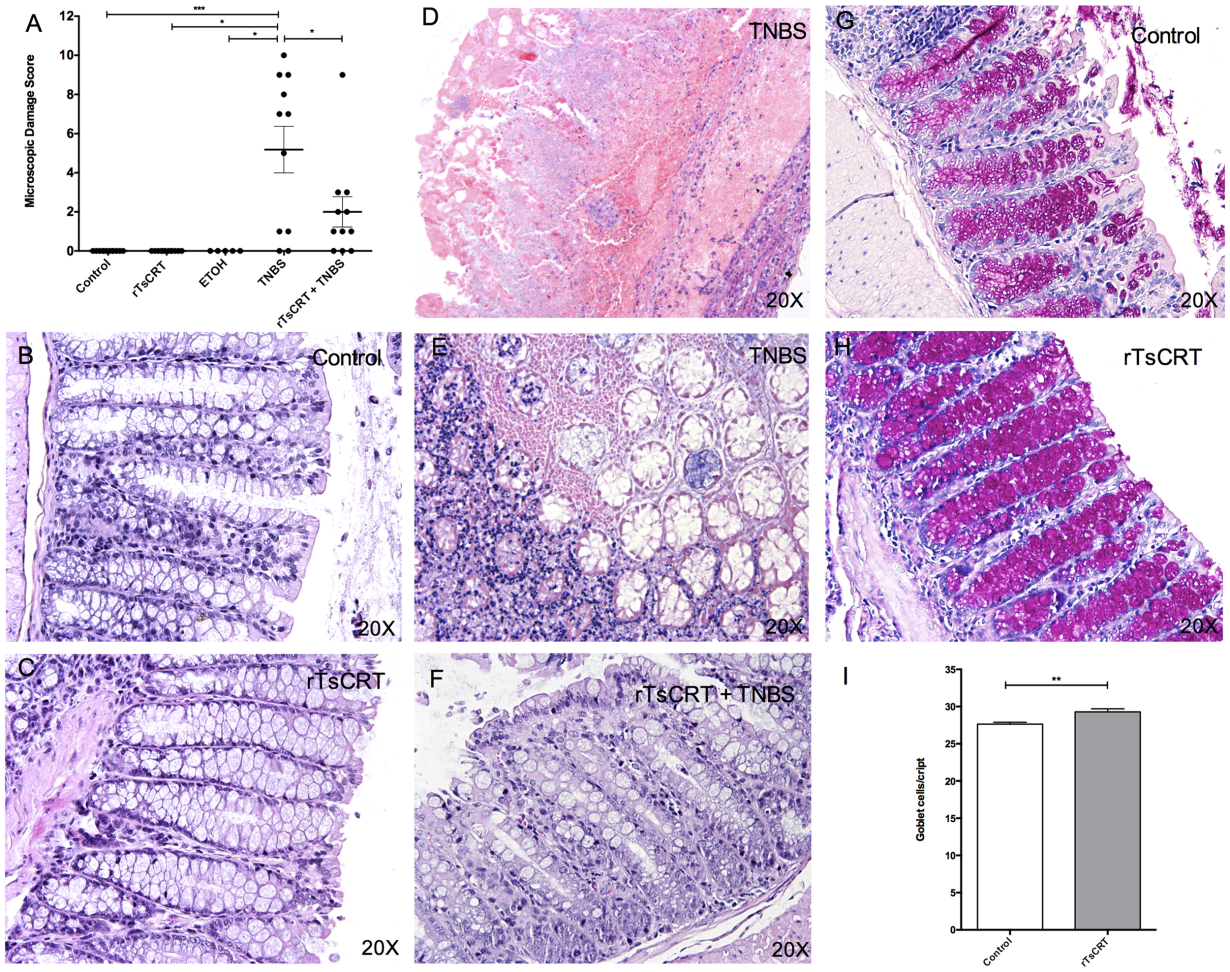
Effect of rTsCRT oral immunization on histological parameters. Microscopic disease score, n = 11 mice per group except in the ethanol treated group (A) and histological observations of colon sections stained with haematoxylin-eosin (20X) of the different experimental groups (B-F). Control (B) and rTsCRT-immunized mice (C) did not show signs of inflammation whereas TNBS-treated mice showed important blood and leukocyte infiltrate, as well as destruction of mucosal architecture (D, E). rTsCRT oral immunization prior to TNBS instillation resulted in histological findings similar to those observed in control mice (F). Representative sections of colons stained with Periodic Acid Schiff (20X) from control (G) and rTsCRT-immunized mice (H). Number of goblet cells per crypt from at least 5 crypts per mouse (n = 12,13) in the control compared with the rTsCRT-immunized mice (I). The Kruskal-Wallis test was used to analyze microscopic damage score (A) and Mann Whitney for goblet cell numbers/crypt (I). Results are expressed as means ± SEM of pooled data from two separate experiments. *P<0.05; **P<0.01; ***P = 0.001.

TNBS induced significant colonic damage as evidenced by the increase in microscopic damage score characterized by polymorphonuclear infiltrate, loss of architecture, edema and number of affected mucosal layers. rTsCRT immunization significantly prevented the development of microscopic inflammation (Fig 2A). The images presented are representative of 11 sections analyzed in each experimental group (Fig 2B–2F). Control mice showed no signs of microscopic inflammation. The structure of the large intestine was normal since the epithelial cell layer was intact, as well as the crypts, and the distribution and characteristics of goblet cells (Fig 2B and 2H). The structure of the colon of rTsCRT-treated mice remained normal (Fig 2C) and a modest but increase in the number of goblet cells and mucus was observed (Fig 2G and 2I). Colons from TNBS-treated mice showed clear signs of inflammation with important blood and polymorphonuclear infiltrates as well as destruction of the architecture, as seen in longitudinal and transverse sections respectively (Fig 2D and 2E). rTsCRT administration prevented the damage produced by TNBS treatment (Fig 2F), in line with the observed suppression of macroscopic and disease parameters in the rTsCRT-immunized mice. Ethanol treatment alone did not result in microscopic damage of the colon (Fig 2A).

### Effect of oral immunization with rTsCRT on pro-inflammatory and anti-inflammatory cytokines in mice with TNBS-induced colitis

**Fig 3:**
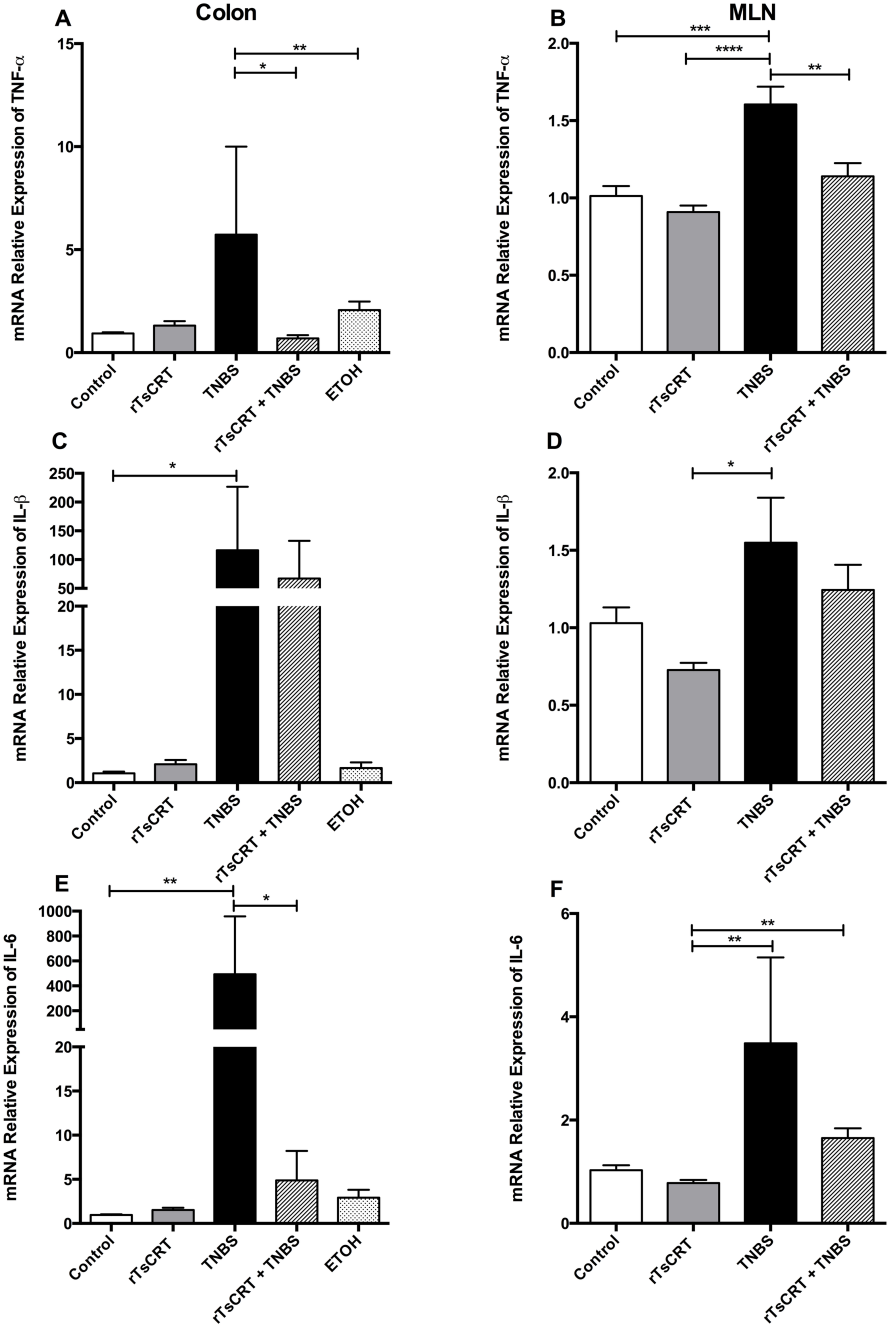
Proinflammatory cytokine mRNA expression in colon and MLN determined by qRT-PCR. mRNA levels of TNF-α in colon (A) and MLN (B); IL-1β in colon (C) and MLN (D) and IL-6 in colon (E) and MLN (F). Data are expressed as the mean of the ratio of each cytokine relative to β-actin (housekeeping gene). Open bars represent data from control mice; grey bars from mice orally immunized with rTsCRT; black bars from mice treated with TNBS; hatched bars from mice orally immunized with rTsCRT and treated with TNBS and dotted bars from mice treated with ethanol. Kruskal-Wallis test was used to analyze relative expression in colon and one-way ANOVA for MLN. Results are expressed as means ± SEM of pooled data from two separate experiments; n = 12,13 mice per group. *P<0.05; **P<0.01; ***P = 0.001.

To analyze the possible association of the prophylactic effect of rTsCRT with cytokine profiles, we analyzed the relative mRNA expression of different cytokines in colon and MLN samples of the different groups of mice. As shown in Fig 3, TNBS instillation significantly augmented the expression of TNF-α, IL-1β and IL-6 in the colonic mucosa and MLN, while oral immunization with rTsCRT prior to TNBS treatment prevented the up-regulation of TNF-α and IL-6 expression in both organs (Fig 3A, 3B, 3E and 3F). Although the lower IL-1β expression levels in the rTsCRT+TNBS group did not reach statistical significance, a clear tendency was observed (Fig 3C and 3D). Ethanol or rTsCRT treatment alone did not increase pro-inflammatory cytokine levels.

**Fig 4:**
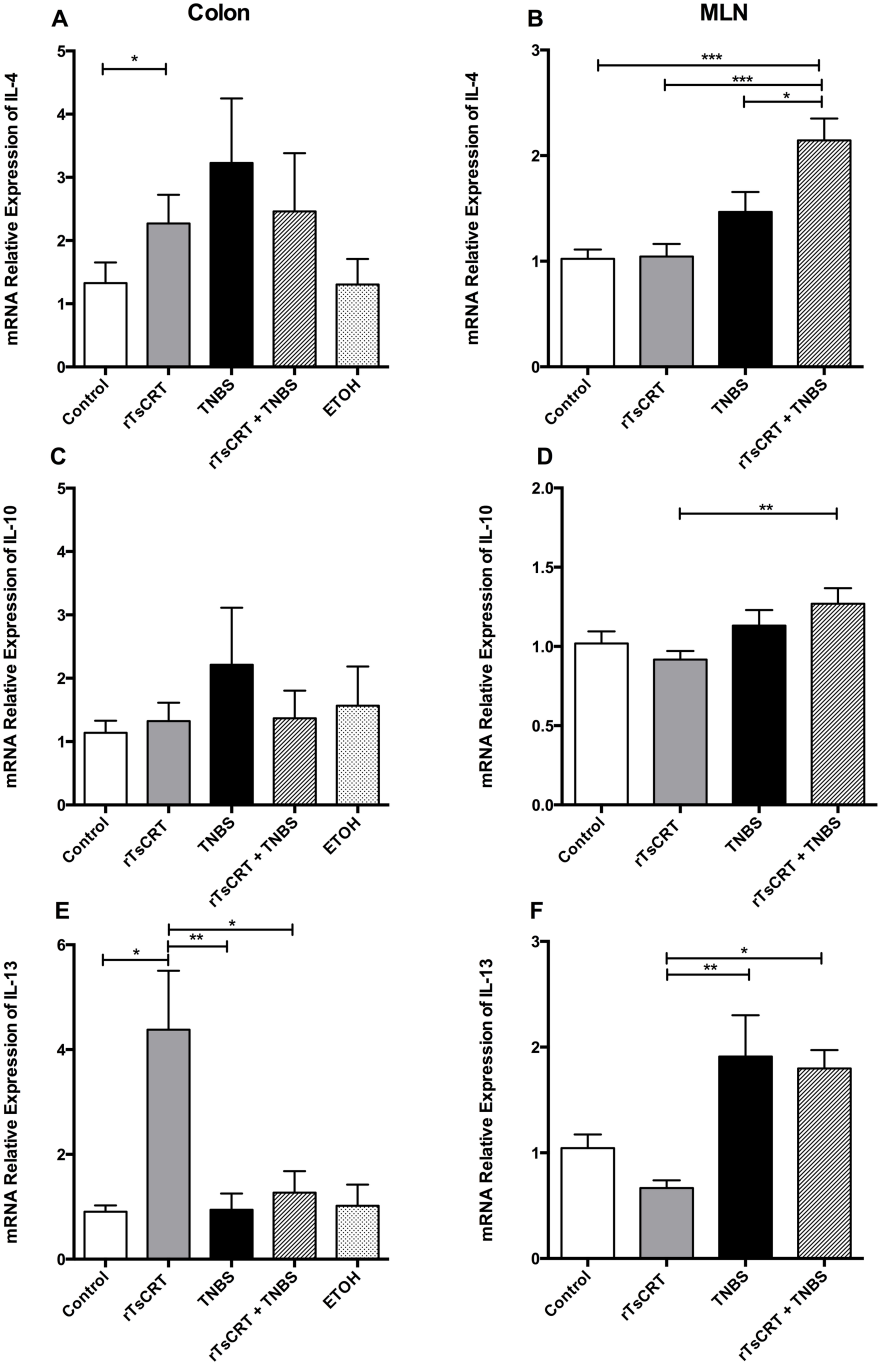
Antiinflammatory cytokine mRNA expression in colon and MLN determined by qRT-PCR. mRNA levels of IL-4 in colon (A) and MLN (B); IL-10 in colon (C) and MLN (D) and IL-13 in colon (E) and MLN (F). Data are expressed as the mean of the ratio of each cytokine relative to β-actin (housekeeping gene). Open bars represent data from control mice; grey bars from mice orally immunized with rTsCRT; black bars from mice treated with TNBS; hatched bars from mice orally immunized with rTsCRT and treated with TNBS and dotted bars from mice treated with ethanol. Kruskal-Wallis test was used to analyze relative expression in colon and one-way ANOVA for MLN. Results are expressed as means + SEM of pooled data from two separate experiments; n = 12,13 mice per group. *P<0.05; **P<0.01; ***P = 0.001.

**Fig 5:**
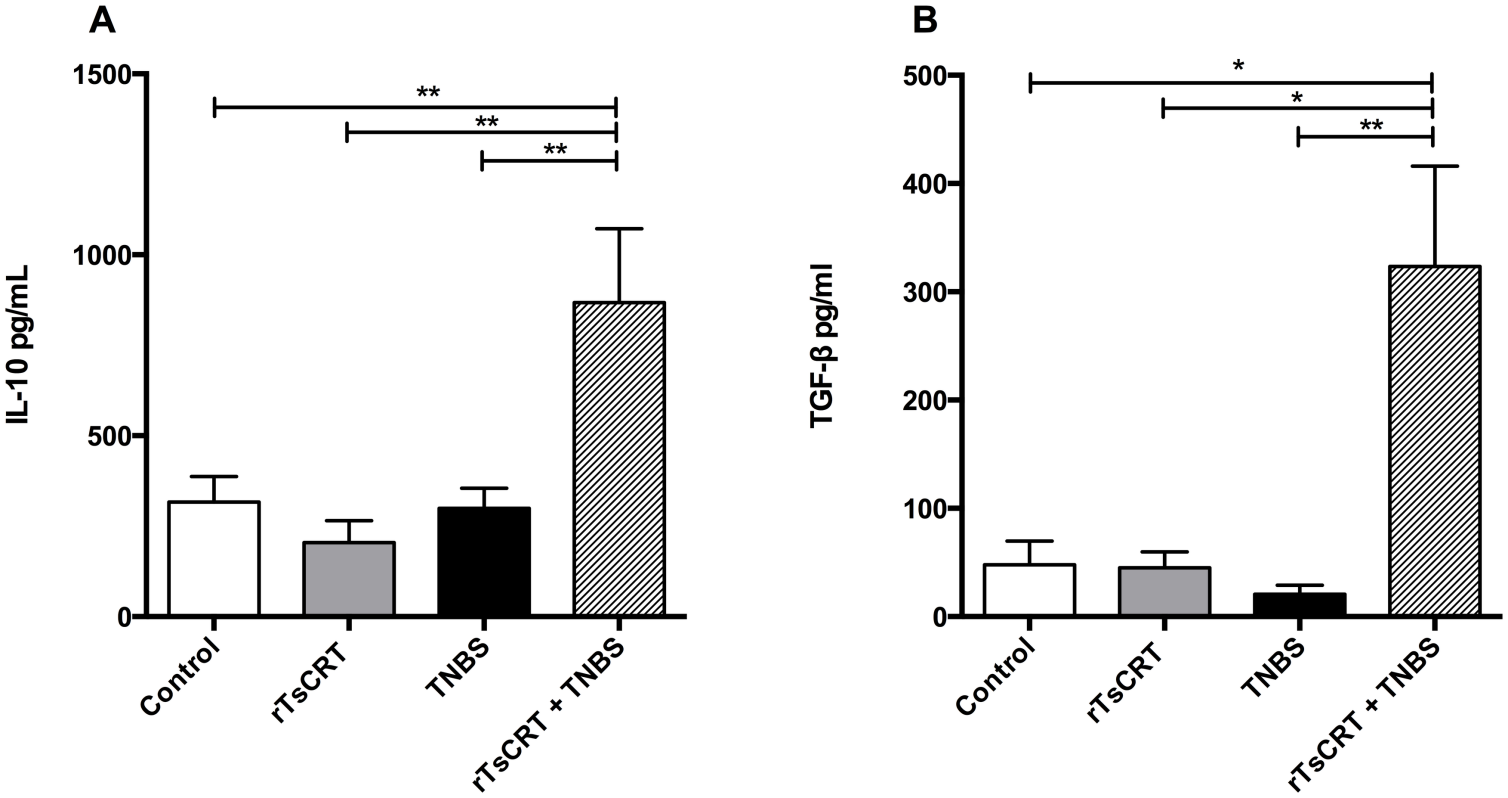
Regulatory cytokine levels in serum. ELISA was used to detect the regulatory cytokines IL-10 (A) and TGF-β (B) in serum samples from the different treated groups. Results are expressed as means ± SEM of pooled data from two separate experiments; n = 12,13 mice per group. Kruskal-Wallis test; *P<0.05; **P<0.01.

Fig 4 shows the mRNA relative expression of anti-inflammatory cytokines in colon and MLN. IL-4 expression in colon was increased in all groups (Fig 4A) and in the MLN of rTsCRT and TNBS treated mice (Fig 4B). IL-10 expression was up regulated in MLN of rTsCRT-immunized mice after induction of colitis but not in colon (Fig 4C and 4D). Interestingly, in rTsCRT-treated mice, a significant over expression of IL-13 was observed (Fig 4E). IL-13 expression was up regulated in the MLN of the two groups that received TNBS compared with the rTsCRT-immunized mice (Fig 4F). IL-10 and TGF-β levels were increased in the serum of mice immunized with rTsCRT and treated with TNBS, as detected by ELISA (Fig 5A and 5B).

### rTsCRT oral administration prevents DNA damage in blood induced by TNBS treatment

**Fig 6:**
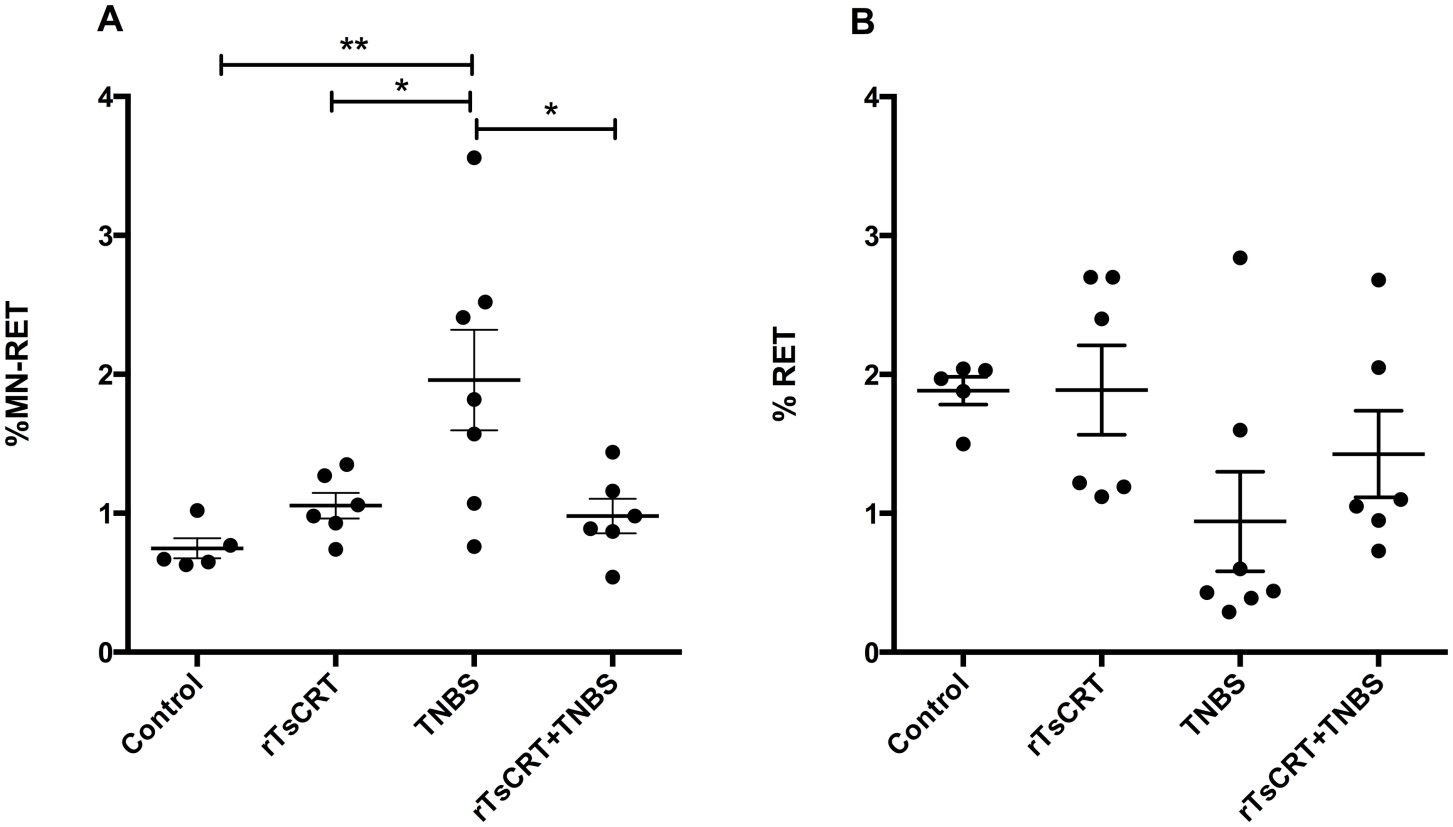
Prevention of genotoxicity induced by TNBS treatment by rTsCRT immunization. Percentage of micronucleated reticulocytes (% MN-RET) as a measure of genotoxic damage (A) and percentage of RET (% RET) as a measure of cytotoxicity (B) in peripheral blood of the different experimental groups. Results are expressed as means ± SEM of pooled data from two separate experiments; n = 12,13 mice per group. One-way ANOVA; *P<0.05; **P<0.01.

Micronuclei (MN) frequency was determined in peripheral blood reticulocytes (RET), as a marker of recently induced chromosomal damage (within 24–48 h) in the different groups of mice by flow cytometry. We confirmed that TNBS significantly increased MN frequency and that oral immunization with rTsCRT prior to TNBS instillation prevented the genotoxic effect of TNBS. In addition, we verified that rTsCRT is not genotoxic, since mice receiving rTsCRT alone did not show high MN production (Fig 6A). The percentage of RET was normal in all groups of mice and varied between 1.2 and 2.5%. In the group that received TNBS, a modest decrease in RET was seen, which may represent a minor cytotoxic effect produced by TNBS (Fig 6B).

To determine the relationship between MN formation and disease activity index (DAI), we performed a Spearman’s correlation analysis between the health status and MN frequency. A positive correlation between DAI and MN frequency was observed (r = 0.608; p<0.001). Mice treated with TNBS had a higher frequency of MN and a greater DAI due to the symptoms of inflammation. Moreover, MN frequency positively correlated with the relative expression of the pro-inflammatory cytokines TNF-α (r = 0.4939; p<0.05), IL-1β α (r = 0.4933; p<0.05) and IL-6 α (r = 0.4357; p<0.05).

## Discussion

Experimental and epidemiological studies have shown that exposure to intestinal helminths protect against the development of diseases that result from a dysregulated immune system, i.e. autoimmune and inflammatory diseases, as well as allergy [1]. The ability to induce type 2 responses shared by several helminths is characterized by a strong regulatory component [34]. Infection or treatment with E/S products from nematodes, trematodes and cestodes ameliorates experimental colitis and other immune-mediated disorders, thus suggesting that helminths share a common strategy to control the immune system of their hosts [1, 4, 5]. The individual molecules responsible for these effects are just beginning to be identified and may differ in their mode of action aiming at different target cells and molecules.

We previously showed that oral administration of rTsCRT induced a predominantly type 2 response that conferred partial protection against *T*. *solium* taeniasis and was able to stimulate the expression of regulatory cytokines during taeniosis in an experimental hamster model [21, 23]. In addition, rTsCRT mainly induced the type 2 cytokines IL-4 and IL-5 as well as specific anti-rTsCRT IgG1, as a result of oral immunization in mice [24]. Here we show that preventive oral administration of rTsCRT protects mice from TNBS-induced colitis, measured by progressive weight loss and disease activity index. In conjunction, the histologic parameters and the macroscopic analysis reflect a significantly less acute colonic inflammation and disruption of the intestinal architecture, confirming the anti-inflammatory nature of rTsCRT. The beneficial effects of rTsCRT oral immunization were associated by the induction of a type 2 and regulatory cytokine expression at the local and systemic levels.

Cytokines are essential mediators of IBD pathogenesis. Pro-inflammatory acute phase cytokines, as well as chronic phase cytokines are key components in the initiation and propagation stages of IBD respectively, while regulatory cytokines mediate the resolution of inflammation [35]. As rTsCRT proved to be beneficial in preventing inflammation, we investigated the cytokine profiles induced as a result of oral immunization. TNF-α, IL-1β and IL-6 are acute phase cytokines, mainly produced by intestinal monocytes and macrophages, involved in the pathogenesis of IBD [36–38]. In the present study we showed that induction of colitis with TNBS caused an up-regulation of these pro-inflammatory cytokines that were significantly diminished by rTsCRT administration. This effect was accompanied by increased expression of the anti-inflammatory cytokines IL-4 and IL-10 in MLN, while IL-13 expression was up-regulated in the colon of rTsCRT-immunized mice, suggesting a modulatory effect of rTsCRT at the induction and effector gut mucosal sites. This finding is in accordance with our previous observations that *T*. *solium* infection induces expression of type 2 cytokines IL-4, IL-5 and IL-13 at the small intestinal mucosa surrounding the scolex of the tapeworms and with studies showing that calreticulin from the mouse nematode *Heligmosomoides polygyrus* induces expression of IL-4 and IL-10 during infection [20, 39]. In addition, a systemic immune-regulatory state was observed, as reflected by the elevated levels of IL-10 and TGF-β in the sera of rTsCRT-immunized mice after the inflammatory stimulus provided by the treatment with TNBS. The fact that rTsCRT induces expression of immunoregulatory cytokines only after TNBS treatment, suggests that rTsCRT does not induce a generalized immunosuppressive state, an important fact in physiological conditions.

Interestingly, we found a significant increase of IL-13 expression in the colon of immunized mice, suggesting a local effect of rTsCRT. The significant increase of IL-13 in colon, as opposed to MLN could be the result of the recirculation of IL-13-producing cells that are induced in MLN, reaching the colon mucosa as effector cells and activators of other cell populations. IL-13 affects mucus production by goblet cells and synergizes with IL-4 and IL-10 in the alternative activation of macrophages. Both mechanisms may have an impact on the prevention of the inflammatory markers induced during colitis [40–42]. Mucus production by goblet cells prevents bacteria from contacting the epithelial cells of the intestinal mucosa and mice that lack mucin production develop severe colitis [43, 44]. The goblet cell hyperplasia observed in our studies after rTsCRT immunization, argues in favor of a protective role for IL-13 by enhancing the mucus barrier function. Concerning the alternative activation of macrophages, preliminary studies using rTsCRT-treated macrophages indicate that the Arg-1 mRNA levels are induced upon rTsCRT treatment (Diaz-Gandarilla, unpublished results).

Severe bowel inflammation is usually accompanied by an increase in DNA damage due to the presence of inflammatory cytokines, reactive oxygen and nitrogen species [13, 45]. Free radicals produced during the inflammatory response can react with DNA and cause rupture of the double helix, increasing the possibility of MN formation [46]. TNF-α, IL-1β and IL-6 induce DNA damage in several cell types [14, 47, 48]. In particular, intestinal inflammation has been shown to elicit systemic genotoxicity that correlates with clinical symptoms as well as pro-inflammatory cytokine levels [13]. Here we showed that rTsCRT administration prevented the genotoxicity induced by TNBS, likely by precluding the expression of the pro-inflammatory cytokines. Consistent with these findings, a positive correlation between MN frequency and relative expression of these cytokines, as well as disease activity index was observed. Accordingly, we have previously reported the reduction of genotoxic damage by rTsCRT in an experimental model of taeniosis [22].

It has been proposed that the most likely mechanism for the amelioration of inflammation is the release of helminth-derived regulatory molecules that are able to modulate different components of the immune system [5]. These molecules evolved as a means for parasites to survive in their hosts and cause minimal immunopathology. For example, cystatins from nematodes and trematodes protect from inflammatory diseases mainly by the induction of regulatory macrophages [49]. More recently, P28GST a recombinant enzyme from schistosomes was shown to prevent colitis though a type 2 response characterized by the presence of modulatory eosinophils [50]. Our findings add a novel immunomodulatory cestode protein to the helminth-derived-synthetically-engineered molecules with anti-inflammatory properties. To our knowledge, calreticulins from *H*. *polygyrus*, *Necator americanus* and *T*. *solium* [20, 21, 23, 24, 51] have Th2 skewing potential in contrast to human and mouse calreticulins, which induce pro-inflammatory cytokines [52, 53]. Whether the ability of calreticulin to promote type 2 responses is a conserved property only of helminth parasites is an interesting issue and warrants additional research.

Correct development, function and regulation of the immune system relies on both internal and external factors, such as environmental and microbial interactions, that have a profound contribution on the balance between health and disease [54]. Helminth exposure is an important element in the regulation of the immune system. Infected individuals display immunologic tolerance since helminth infections are able to modify several pathways of immune activation, that include dendritic cell or macrophage stimulation, cytokine and antibody production, polarization of T and B cell populations, among others [5, 55]. *T*. *solium* is particularly effective in regulating pathologic inflammation responsible for the symptoms of the disease, considering that both live cysticerci and the intestinal tapeworm do not cause overt symptomatology [56]. We propose that rTsCRT mimics the beneficial effects on the immune system that result from exposure to intestinal helminths. Identifying the regulatory molecules involved in such processes could translate into the development of new therapeutics for the prevention of acute inflammation that characterizes the onset of IBD, where innate immune mechanisms play an important role. In the present paper we used an acute model of colitis induced by TNBS that does not reflect the chronicity of the human disease. Nevertheless, this model simulates some of the cytokine profiles and histopathological features of IBD and is a useful tool for studying the underlying mechanisms and potential therapeutics [57, 58]. Further studies are necessary to assess the effects of rTsCRT in a chronic model of colitis that more closely mimics the exacerbation episodes characteristic of the remission-relapse cycles in human IBD. Treatment with rTsCRT during remission periods could also be useful in the prevention of flare-ups. We are currently using a chronic model of colitis to explore this possibility.

In conclusion, oral immunization with rTsCRT prevents severe clinical symptoms and intestinal inflammation. The anti-inflammatory effect of rTsCRT is associated with type 2 and regulatory cytokine responses characterized by colonic IL-13 and systemic IL-10 and TGF-β. Further analysis of the molecular mechanisms by which rTsCRT precludes the inflammatory processes driven by dysregulated cytokine circuits as well as the mechanisms involved in the prevention of DNA warrant additional research.
